# Increased Upper Extremity Muscle Mass in Ambulatory Children with Cerebral Palsy

**DOI:** 10.3390/life14030303

**Published:** 2024-02-26

**Authors:** Taeyoung Song, Jaewon Kim, Dae-Hyun Jang

**Affiliations:** Department of Rehabilitation Medicine, Incheon St. Mary’s Hospital, College of Medicine, The Catholic University of Korea, Seoul 06951, Republic of Korea; tokee2@naver.com

**Keywords:** cerebral palsy, muscle mass, dual-energy X-ray absorptiometry, lean body mass, Gross Motor Function Classification System

## Abstract

Aim: To compare muscle mass in the upper and lower extremities between ambulatory children with cerebral palsy (CP) and typically developing (TD) children. Materials and Methods: A total of 21 children aged 2 to 12 years with CP and a Gross Motor Function Classification System (GMFCS) level of I, II, or III were matched with 21 TD children for age, sex, and body mass index. The lean body mass (LBM) of each extremity was calculated from whole-body dual-energy X-ray absorptiometry. Results: The LBM of the upper extremities was greater in children with CP compared to TD children, and the difference was significant in the GMFCS level II group (1340.6 g vs. 1004.2 g, *p* = 0.027). There was no significant difference in the LBM of the lower extremities between the CP and TD groups (*p* = 0.190). The ratio of lower extremity LBM to total extremity LBM was lower in children with CP, while the ratio of upper extremity LBM to total extremity LBM was higher in children with CP (73.2% vs. 78.5% [*p* < 0.001] and 26.7% vs. 21.5% [*p* < 0.001], respectively). Conclusions: Ambulatory children with CP, especially in the GMFCS level II group, exhibit greater muscle mass in the upper extremities compared to TD children.

## 1. Introduction

Cerebral palsy is a prevalent childhood physical disability worldwide, occurring at a rate of 2–3 per 1000 individuals [1]. It is a group of disorders that affect movement, muscle tone, and posture, causing muscle weakness, spasticity, or dystonia [1,2]. It is well established that the muscle mass of the lower extremities in children with CP is reduced compared to typically developing (TD) children from previous studies [3,4,5,6,7,8,9,10,11]. The reduction in muscle mass is attributed to the functional difficulties in lower extremity use during activities like walking, arising from factors such as spasticity. Likewise, children with CP experience restricted functional movement in the upper extremities [12]. Nevertheless, ambulatory children with CP find themselves in an environment where they rely relatively more on their upper extremities in daily activities. In the conflicting situation between upper limb dysfunction and the use of upper extremities for daily activities, there has been little research on the muscle mass of the upper extremities to date. Due to the lack of such studies, it is still unclear how upper extremity muscle mass differs between TD children and ambulatory children with CP.

In previous studies, muscle mass in children with CP has been measured using several modalities [5,6,7,8,9,10,11]. Among these techniques, magnetic resonance imaging (MRI) has demonstrated high accuracy and reproducibility [5,6]. However, MRI measures have some limitations. First, to measure the masses of most or all extremity muscles, the child must maintain a specific position for several tens of minutes, which often requires sedation. Additionally, MRI examinations are costly and, in many cases, the examination itself can be burdensome to the family. For these reasons, most such studies on children with CP have concentrated on measuring the masses of major muscles in the lower extremities, such as the gastrocnemius, soleus, and rectus femoris, and few studies have examined the muscle masses of both lower and upper extremities in children with CP [5,6,7,8,9,10,11]. Ultrasound, on the other hand, offers the advantage of being able to measure various muscles without the requirement for sustained postures. However, it lacks clear criteria for measurement, leading to potential variations in results depending on the examiner. Additionally, the time and cost involved in measuring the entire set of muscles are considerable, diminishing efficiency. Despite these challenges, understanding the entirety of muscle mass distribution is crucial for gaining a comprehensive insight into the impact of CP on the musculoskeletal system. 

An alternative method with several advantages for comprehensive quantitative measurement of the entire skeletal muscle mass in both upper and lower extremities is dual-energy X-ray absorptiometry (DXA). This method enables the quantitative measurement of whole-body mass including muscle tissue, blood vessels, connective tissue, fat cells, and bone. It also provides a measurement of regional lean body mass (LBM) by excluding fat and bone tissue, focusing on tissues that do not include muscles [13,14,15,16,17,18]. For this reason, DXA can overestimate skeletal muscle mass when compared to skeletal volume measured by MRI [16,19]. However, recent studies investigating the accuracy of measuring muscle mass with DXA in children with CP have reported strong correlations with MRI measures [13,16,20,21]. This correlation is substantial enough that DXA is currently employed in evaluating sarcopenia in adults. Furthermore, it has been shown to be a reliable indicator of skeletal muscle mass measurement in children and adolescents [13].

The hypotheses of this study are twofold. First, based on previous research indicating a reduction in local muscle mass in the lower extremities compared to TD children when measured through MRI in children with CP who have functional limitations in the lower extremity, we anticipate consistent results when measuring overall lower extremity muscle mass using DXA. Second, we suggest that independently ambulant children with CP, despite exhibiting impairments in upper extremity function, may not experience as significant a reduction in upper extremity muscle mass compared to their lower extremities. This assumption stems from the observation that these children, necessitating increased activity in daily living, might retain or even develop muscle mass in the upper extremities to compensate for their functional limitations.

The primary objective of this study is to quantitatively assess the LBM of both the upper and lower extremities in ambulatory children with CP and their TD counterparts. By comparing the LBM in these distinct groups, we aim to substantiate the aforementioned hypotheses. This comprehensive analysis seeks to provide a clearer understanding of how CP affects muscle distribution, potentially guiding more tailored and effective therapeutic interventions. Through this, we investigate whether the reduced motor function in the upper and lower extremities results in lower muscle mass compared to a group of TD children. 

## 2. Materials and Methods

This study is registered as a clinical trial with Clinical Research Information Service (CRIS), Republic of Korea (KCT0008067). This study was conducted in accordance with the ethical standards of the Guidelines for Good Clinical Practice. The goal, procedure, and safety aspects of the study were explained to each participant and their primary caregivers provided written informed consent before their participation.

### 2.1. Participants

Participants were assessed in Incheon Saint Mary hospital affiliated to the Catholic University of Korea between October 2021 and July 2023 for eligibility. They underwent measurements of height and weight, with minimal clothing, and wearing no shoes or assistive devices. Standard measuring tools were employed in these assessments. Body mass index was then derived based on the recorded height and weight.

#### 2.1.1. CP Group

The inclusion criteria for the CP group were as follows: (1) children diagnosed with spastic CP, (2) aged 2 to 12 years, and (3) Gross Motor Function Classification System (GMFCS) level of I to III [22]. The exclusion criteria were (1) children with a history of orthopedic surgery, (2) children receiving botulinum toxin injections within the past 6 months, and (3) children with concomitant neurological disorders other than CP.

#### 2.1.2. TD Group

Children aged 2 to 12 years demonstrating normal gross motor development were recruited. Inclusion criteria were (1) ability to walk independently before 15 months of age, (2) normal muscle tone, and (3) children classified as ‘Normal’ on the Korean Developmental Screening Test [23]. We matched the CP group and TD group based on age within 12 months, difference of height and weight within 10%, and body mass index within 1.0 kg/m^2^. The reason for stringent matching was to account for the broad age range of the participants, recognizing that the outcomes could vary widely within this age range, and to ensure that the matching considered the diverse developmental situations, minimizing the potential impact of age-related variations in body composition and muscle mass.

### 2.2. Gross Motor Function Assessment

The GMFCS level of participants in the CP group was assessed by the primary physician using the extended and revised version of the GMFCS released in 2007, while considering developmental milestones and primarily categorizing the participants into the age ranges of 2 to 4 years, 4 to 6 years, and 6 to 12 years [22,24]. Focusing on the most prevalent age group, which is 6 to 12 years, individuals at GMFCS level I can walk without restrictions. For GMFCS level II, children require physical assistance in activities such as walking, and they need a handrail when ascending stairs. GMFCS level III typically involves walking indoors with the aid of hand-held walking devices [22]. To evaluate upper extremity function, the Manual Ability Classification System (MACS) was also used for assessing participants in the CP group, applying consistent criteria regardless of age [24,25]. 

### 2.3. DXA Measurement

**Figure 1:**
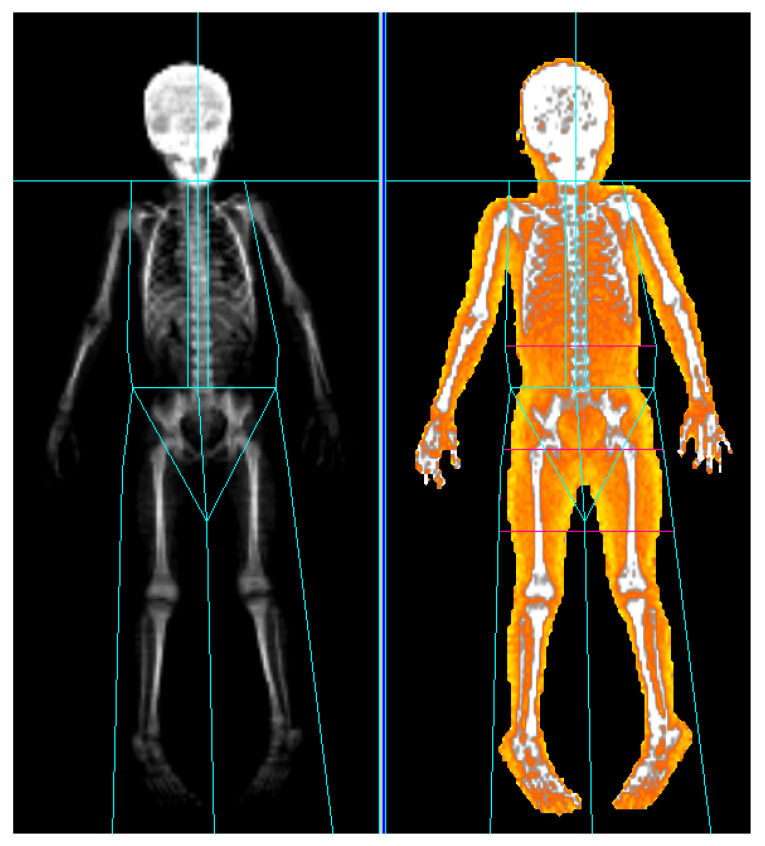
Total body mass assessment by dual-energy X-ray absorptiometry. In the example illustrated, the total body mass and fat mass of a 5-year-old child with CP were measured and divided into upper and lower extremity parts according to the indicated landmarks using Encore version 14.1 (GE Healthcare).

The measurement procedures were similar to those described in our previous study [18]. Whole-body DXA scans were conducted in the supine position, ensuring the inclusion of the entire body. Participants were instructed to extend their arms to the side sufficiently to allow clear separation from the trunk, and to keep their legs apart to avoid any contact. They were instructed to adopt a position where the palms and soles faced the ground while ensuring that the body did not rotate. During the scanning process, patients were instructed to minimize voluntary muscle contractions and not to correct their posture for several minutes. A familiar caregiver was present beside them. Restraint mats were employed for patients facing challenges in maintaining their posture. We obtained scan results for all participants using the Prodigy densitometer (GE Healthcare, Madison, WI, USA). The LBM of the upper and lower extremities was determined by subtracting the mass of fat and bone tissues from the total tissue mass using Encore version 14.1 software (GE Healthcare) [13,14,15,16,17,18]. The initial partitioning was automatically generated by the software, dividing the body into parts such as the head, both arms, the trunk, both legs, and gonads. For precise partitioning, manual adjustments were made by the technician. Anatomical landmarks were utilized for this purpose. For the measurement of the lower extremities, a line was drawn perpendicular to the axis of the femoral neck, defining the range from this line to tips of the toes. For the upper extremities, a line was drawn connecting the humeral articulation in both shoulder joint sockets, defining the range from this line to the fingertips (Figure 1).

### 2.4. Statistical Analysis

All statistical analyses were performed using IBM SPSS (version 29.0, IBM Corporation, Armonk, NY, USA). Group variables and extremity LBM are expressed as mean (±standard deviation). Baseline clinical characteristics, including age, height, body weight, body mass index, extremity LBM, and the LBM ratio for total extremities, were compared between groups using independent *t*-tests to identify any significant differences. Additionally, LBM results were categorized by GMFCS level and compared between upper and lower extremities within each level through independent *t*-tests to ascertain differences. For subgroup analyses, we assessed LBM differences between affected and unaffected sides among unilateral CP patients and TD children. In the TD group, each side was analyzed separately—effectively considering one individual as two entities—and then compared with the CP group. A *p* < 0.05 was considered statistically significant.

## 3. Results

### 3.1. Participant Matching and Clinical Characteristics

The 21 children in the CP group were matched for age, sex, and body mass index with 21 TD children (10 males and 11 females each). There were no significant differences in mean age, height, body weight, and body mass index between the two groups (Table 1). In the CP group, all children were classified as ambulatory according to the GMFCS by age, with 13 children at level I, 5 at level II, and 3 at level III. Additionally, the MACS scores indicated that all children were reasonably adept at manual tasks, with 17 children at level I and 4 at level II. Moreover, 8 patients exhibited unilateral and 13 showed bilateral subtypes. Eleven children had received botulinum toxin injection treatment (but not in the previous 6 months), with eight having received a single injection and three having received two injections. They all underwent injections in the lower extremities, with six children receiving injections on both sides and five receiving injections on one side only (Table 2).

### 3.2. Comparisons of Extremity LBM

The LBM of upper, lower, and total extremities and the ratio of upper and lower extremity LBM to total extremity LBM are presented in Table 3. The LBM of the upper extremities was significantly greater in the CP group than the TD group (mean [SD]: 1330.9 [403.2] g vs. 1112.6 [316.3] g, *p* = 0.029). The LBM of the lower and total extremities did not differ significantly between groups. However, the ratio of lower extremity LBM to total extremity LBM was significantly smaller in the CP group compared to the TD group (73.2 [4.1] % vs. 78.5 [2.2] %, *p* < 0.001), while the ratio of upper extremity LBM to total extremity LBM was significantly greater in the CP group (26.7 [4.1] % vs. 21.5 [2.2] %, *p* < 0.001). Table 4 presents the LBM values for each upper and lower extremity by GMFCS level, as well as the ratio for the total extremities. Significant differences in upper extremity LBM were observed between groups at level II (1340.6 [151.0] g vs. 1004.2 [296.8] g, *p* = 0.027), while no significant differences were observed at levels I and III. The lower extremity LBM did not show differences across all levels. However, the ratio of extremity muscle mass to total extremity muscle mass showed significant differences between groups at all levels.

Table 5 presents a comparative analysis of the LBM in the upper and lower extremities between the unilateral CP group (*n* = 8), distinguishing between affected and unaffected sides, and the TD group. Although the LBM of the unaffected side’s upper extremity was relatively greater than that of the affected side, there was no statistical significance when compared to the children with TD [612.4 g (affected) versus 649.8 g (TD) and 658.9 g (unaffected) versus 640.0 (TD)]. Likewise, for the lower extremity LBM, no significant differences were found when compared to the TD group on either side.

## 4. Discussion

In the present study, we quantitatively compared the upper and lower extremity muscle mass of children with CP using DXA, matched for gender, age, and body mass index with TD children. We utilized LBM calculated from DXA measurements as a method for quantification. The LBM of the upper extremities was higher in children with CP compared to TD children, and the difference was significant in the GMFCS level II group. There was no significant difference in the LBM of the lower extremities between the CP and TD groups. The ratio of upper extremity LBM to total extremity LBM was significantly higher in the children with CP, while the ratio of lower extremity LBM to total extremity LBM was significantly lower across all GMFCS levels in the CP group. 

As hypothesized, the upper extremity muscle mass in the CP group did not decrease compared to the TD group. Instead, the significant increase in upper extremity LBM observed in children with CP in our study contradicts expectations based on previous research that measured various upper extremity muscle strengths [12]. In the study conducted by Dekkers et al., it was observed that children with CP generally exhibited weaker upper extremity strength than TD children, but their specific muscle strength, including elbow flexors and extensors on their preferred side, was significantly higher than that of TD children. However, they did not take GMFCS levels into account, which imposes limitations on the correlations with our study [12]. Notably, when stratified by GMFCS level, a significant increase in LBM was observed at level II. The reasons for this are, first, that all children with GMFCS levels of II or III who participated in this study (8 out of 21) were wearing a foot and/or ankle orthosis, and 4 were using a walker for ambulation support (3 individuals walked using walkers, while 1 5-year-old boy used a walker only when going out and walked independently indoors). Second, children with GMFCS level II may particularly use their upper extremities during activities, such as using railings while ascending and descending stairs or pushing against a stable surface when transitioning from a seated to a standing position, which require the application of isotonic force [1,22]. In addition to basic mobility, they need to grasp or push desks, shelves, handles, and other assistive devices during daily activities such as tooth brushing, dressing, object manipulation, and maintaining balance while standing, demanding a continuous application of isometric force. At GMFCS level III, walking aids or canes are commonly used for ambulation support. During these activities, weight-bearing exercises are frequently performed, but there is a relatively higher dependency on assistive devices for daily movements. Daily activities are often performed while sitting, and assistance from others is required when climbing stairs. Consequently, the frequency of isometric exercise may decrease, potentially leading to less significant gains in upper extremity muscle strength. Indeed, among the three children classified as GMFCS level III, only one was categorized as MACS level II. In the GMFCS level II group, two out of five children were classified as MACS level II, while the remaining three were at MACS level I [25]. 

Among the children with CP who participated in our study, a larger proportion exhibited bilateral CP. Individuals with unilateral CP possess one unaffected upper extremity, potentially contributing to greater independence in daily activities. Therefore, it was crucial to distinguish between the affected and unaffected sides when evaluating upper extremity muscle mass. Consequently, the LBM of the unaffected side in the children with unilateral CP was numerically greater compared to the TD children, consistent with findings observed in the lower extremities. These results suggest that children with unilateral CP may rely more on the unaffected side during ambulation and daily activities. However, due to the small sample size, statistically significant results were not observed.

Increased muscle mass in the upper extremities has important therapeutic implications, particularly for children with spastic CP receiving botulinum toxin injections, as these treatments can reduce muscle mass, thereby impairing activities dependent on greater upper extremity strength [3,26,27]. Our recent study revealed that the muscle mass decrease caused by botulinum toxin injections does reverse over the long term, but patients may experience difficulty during the recovery period [18]. Nonetheless, upper extremity botulinum toxin therapy for spasticity treatment may pose less of a burden compared to lower extremity treatment. A similar rationale could be applied to Constraint-Induced Movement Therapy. Constraint-Induced Movement Therapy is commonly employed not only for stroke patients but also for children with unilateral CP to improve the strength and functionality of the affected hand [28,29]. However, due to the constraint of the unaffected hand’s movement, it might induce alterations in the muscle mass of the unaffected upper extremity. In this regard, the findings of this study could be utilized in therapeutic planning. The observed increase in muscle mass in children classified as GMFCS level II has significant implications for guiding therapeutic strategies. This finding supports the rationale for prioritizing goal-directed or task-specific interventions over traditional muscle-strengthening therapies, such as electrical stimulation or vibration therapy. By focusing on activities that are directly related to daily functions and individual goals, therapy can be more effectively tailored to enhance upper extremity function, thereby potentially improving the quality of life and independence of children with CP [30].

Contrary to our initial hypothesis, which focused on comparing the LBM reflecting lower extremity muscle mass, we found no significant differences in absolute values between the CP and TD groups. This was consistent across all GMFCS levels and might be attributed to the strict matching of gender, age, and body mass index between groups, as well as the fact that all children with CP in the study had sufficient lower extremity function for walking compared to the TD children, thus minimizing significant differences. However, the ratio of lower extremity LBM to total extremity LBM showed a significant decrease when corrected for the matched variables, indicating a reduction in lower extremity muscle mass. This is in line with previous studies comparing muscle volume in children with CP. 

The present study has several strengths. There are few existing studies in CP research that involve a one-to-one matching with TD children. However, by strictly matching for age, height, and body mass index, we aimed to minimize factors that could introduce errors into the interpretation of the results, such as individual age and weight differences [5,13]. Additionally, in contrast to previous research that measured the muscle volume of each muscle individually, our study measured the overall muscle mass of extremities, considering the heterogeneity in muscle size within the CP group [5,13,31]. 

There are also limitations in our study. First, the sample size was relatively small due to the requirement for proper positioning during the DXA examination. The children had to lie still for several minutes in a supine position with arms and legs spread, which was challenging for many participants during the test period. To reduce invasiveness, scans were performed without sedation. Consequently, for more than 30% of the enrolled subjects across both the CP and TD groups, we were unable to obtain DXA scan results, resulting in their exclusion from the study population. Furthermore, performing subgroup analyses within the CP group resulted in a reduced sample size, consequently elevating the risk of Type I errors during statistical multiple comparisons. Second, the standard deviations of the results were large in comparison to the mean, a phenomenon attributed to the participants’ wide age range, which spanned from 2 to 12 years. To minimize these issues, we utilized an LBM ratio by dividing each extremity into the total extremity. Third, out of 21 participants in the CP group, 10 had received previous botulinum toxin injections, although due to the minimum 6-month interval specified in the inclusion/exclusion criteria, it was assumed that this interval had no significant influence on the current extremity muscle mass [18,28]. Fourth, the accuracy of the lower extremity measurements was limited by the partitioning method. Specifically, soft tissue above the femoral neck was excluded, which resulted in only a partial measurement of the large gluteal muscles, potentially influencing the absolute values in the lower extremity LBM comparison. 

## 5. Conclusions

Our findings reveal that ambulatory children with CP, particularly those classified as GMFCS level II, had greater muscle mass in the upper extremities compared to their TD counterparts. 

## Figures and Tables

**Table 1:** Baseline clinical characteristics of the study subjects.

Characteristic	CP Group (*n* = 21)	TD Group (*n* = 21)	*p*-Value
Sex, male:female (*n*)	10:11	10:11	
Age (mo)	71.95 (23.19)	69.76 (22.54)	0.433
Height (cm)	111.48 (12.20)	112.04 (12.10)	0.440
Body weight (kg)	20.31 (5.40)	20.23 (5.23)	0.480
Body mass index	16.09 (1.45)	15.88 (1.38)	0.314

Data are mean (SD) unless otherwise indicated. CP, cerebral palsy; TD, typically developing.

**Table 2:** Characteristics of the CP Group Participants.

Characteristic	CP Group (*n* = 21)	
Classification	Spastic bilateral	13
	Spastic unilateral	8
GMFCS level	I	13
	II	5
	III	3
MACS level	I	17
	II	4
Previous botulinum toxin treatment	None	10
	Once	8
	Twice	3

CP, cerebral palsy; GMFCS, Gross Motor Function Classification System; MACS, Manual Ability Classification System.

**Table 3:** Lean body mass of extremities analyzed by dual-energy X-ray absorptiometry.

Variables	CP Group	TD Group	*p*-Value
Lean body mass (g)			
Upper extremities	1330.9 (403.2)	1112.6 (316.3)	0.029
Lower extremities	3740.7 (1376.7)	4108.1 (1304.6)	0.190
Total extremities	5072.8 (1722.8)	5220.6 (1603.0)	0.387
Ratios of lean body mass (%)			
Upper/total extremities	26.7 (4.1)	21.5 (2.2)	<0.001
Lower/total extremities	73.2 (4.1)	78.5 (2.2)	<0.001

Data are mean (SD). CP, cerebral palsy; TD, typically developing.

**Table 4:** Lean body mass and ratios of upper and lower extremities to total extremities according to GMFCS level.

Variables	GMFCS I (*n* = 13)	TD (*n* = 13)	*p*-Value	GMFCS II (*n* = 5)	TD (*n* = 5)	*p*-Value	GMFCS III (*n* = 3)	TD (*n* = 3)	*p*-Value
Upper extremities (g)	1335.8 (493.3)	1176.9 (335.1)	0.173	1340.6 (151.0)	1004.2 (296.8)	0.027	1293.7 (343.2)	1014.3 (282.8)	0.169
Lower extremities (g)	4103.69 (1552.8)	4436.00 (1356.8)	0.284	3328.2 (938.1)	3575.0 (1314.0)	0.371	2855.0 (515.5)	3575.3 (827.6)	0.135
Upper/total extremities (%)	24.7 (2.3)	21 (1.6)	<0.001	29.5 (5.5)	22.4 (3.9)	0.023	30.9 (2.2)	22 (1.0)	0.002
Lower/total extremities (%)	75.3 (2.3)	79 (1.6)	<0.001	70.5 (5.5)	77.6 (3.9)	0.023	69.1 (2.2)	78 (1.0)	0.002

Data are mean (SD). GMFCS, Gross Motor Function Classification System; TD, typically developing.

**Table 5:** Comparison of lean body mass between the affected and unaffected sides among unilateral CP and matched TD groups.

Variables	CP (*n* = 8)		TD (*n* = 8)	*p*-Value
Upper extremity (g)	Affected	612.4 (208.48)	649.8 (115.48)	0.314
	Unaffected	658.9 (170.88)	640.0 (117.22)	0.400
Lower extremity (g)	Affected	2037.4 (739.87)	2310.4 (505.41)	0.202
	Unaffected	2226.3 (628.73)	2310.5 (493.38)	0.385

Data are mean (SD). CP, cerebral palsy; TD, typically developing.

## Data Availability

Data are contained within the article.
